# Supplementary material for: Arbovirus-vector protein interactomics identifies Loquacious as a co-factor for dengue virus replication in Aedes mosquitoes
Source: PLoS Pathog. 2022 Sep 8;18(9):e1010329. doi: 10.1371/journal.ppat.1010329 (PMC9488832; doi:10.1371/journal.ppat.1010329)
Supplement: Attachment — Submitted filename: 20220721-Loqs_PLOSpath-Response_to_reviewers_R2_submitted.pdf [file ppat.1010329.s013.pdf]

Nijmegen, July 21, 2022

Manuscript number D-22-00212R1

Dear editor,

We thank the reviewers for their supportive comments on our manuscript "Arbovirus-vector protein interactomics identifies Loquacious as a co-factor for dengue virus replication in *Aedes* mosquitoes".

We have addressed the final comments of Reviewer #2 and revised the manuscript when necessary. Additionally, we noted that the wrong reference genome (*Ae. albopictus*) had been used for our Loqs mass spectrometry analyses in the R1 version of our manuscript. We have repeated the analyses, now using the correct *Ae. aegypti* annotation (Fig. S1 and Tab. S4). Methods were clarified accordingly (lines 477-481 and 491-494). As expected, the new analysis did not affect the results significantly and leads to the same conclusions (lines 193-198).

Please find enclosed the revised manuscript, one version with our changes highlighted in yellow as well as a clean version. Our responses to the reviewers' specific comments can be found below (in blue, italic font).

With best wishes,

Ronald van Rij

Part I - Summary

Reviewer #1: The authors did a very thorough job addressing the reviewers concerns through the addition of more explanatory text, extra experiments and additional controls.

Reviewer #2: The manuscript is now substantially improved, and the authors put quite some efforts in providing important missing controls on EMSA assays and modulation of piRNA. Furthermore, additional clarifications on experimental set-up s and statistical analysis were added, and some statements on interpretation of experiments were tuned down. Nonetheless, this reviewer feels that the claim that Loq plays a role in vRNA replication is still not experimentally supported (see comment below).

Reviewer #3: The authors have put considerable effort into addressing the reviewers' comments with a number of rewrites, additional experiments and analyses. My original comments have been addressed in full and I have no further comments.

We thank the reviewers for their supportive comments. We addressed the points of Reviewer #2 below.

Part II – Major Issues: Key Experiments Required for Acceptance

Reviewer #1: None noted

Reviewer #2: This reviewer is still particularly concerned on the interpretation of the Loq k.d. results on viral RNA replication. Indeed, despite the clarifications provided by the authors, in the revised manuscript there is still no experimental evidence that Loqs plays a role in viral RNA replication.

The justification that in the authors' hands the subgenomic replicon used is "not stable/degraded" over time seems not to be experimentally supported, as data presented are normalized by the 3h R.L.U. value, which represent the input (i.e. translation of in vitro synthesized RNA). Clearly, both in Figure R1 and in Figure 4h, this replicon replicates above input values for at least 24-40h. To my knowledge a viral RNA which does not replicate should decline over time. In vertebrate cells this usually happens at 12h.p.t..

Importantly, the new sentences added in the text do not resolve this issue, but simply state that according to the authors this replicon does not replicate, lines 221-223: "To this end, Renilla luciferase expression was monitored after transfection of subgenomic replicon RNA into Ae. aegypti Aag2 cells, under experimental conditions in which replicon RNA is translated but not replicated (Fig. 4H)". If such claim has to be made, comparing a R.L.U. activity of a replication-deficient replicon (such as an NS5 "GND-mutant" which by definition does not replicate but can be translated) with a replication-competent would be required.

We do not agree with the reviewer's interpretation of our replicon data. As shown by others (Fig. 1D in Alvarez et al., 2015, https://doi.org/10.1016/j.virol.2005.06.009, Fig. 5C in Shivaprasad et al., 2022, https://doi.org/10.1371/journal.ppat.1010163), luciferase counts from DENV or ZIKV replicons accumulate during the first hours after transfection, followed by a sharp increase in luciferase activity after 24 h. In contrast, in our assay luciferase activity of DENV replicon transfected cells accumulate during the first hours after transfection, after which it decreases from 24h onwards. Importantly, this pattern reflects the pattern observed for a replication-defective mutant replicon in Fig. 5C of Shivaprasad et al., 2022 (https://doi.org/10.1371/journal.ppat.1010163), where luciferase counts accumulated during the first hours following the transfection as well, after which counts declined. We therefore stand with our original interpretation that our replicon does not replicate, and therefore with our conclusion that Loqs does not affect viral RNA stability and translation efficiency.

Additionally, also the new experiment provided upon suggestion by Reviewer 3 (Fig4i), does not corroborate the hypothesis that Loq affect viral RNA replication. Indeed, the crucial experiment proposed by reviewer 3 was to "measure the accumulation of negative-sense viral RNA in both the replicon and infection experiment to directly measure viral genome replication."

The experiments provided in new Fig. 4i were performed only with full length viruses in conditions where multiple cycles of replication take place (MOI=0.01, readout by qRT-PCR at 48h.p.i.), thereby confounding/additive effects of viral entry, RNA replication and viral spread on the observed reduction in (-)vRNA cannot be ruled out.

This experiment, or any other unequivocally supporting the hypothesis that vRNA replication is affected is required. Alternatively, the author should rephrase the corresponding paragraphs referring to a more general effect on "viral replication".

Given our results we deem it most likely that Loqs affects viral RNA replication. However, we agree with the reviewer that we have not unequivocally demonstrated that Loqs is required for viral RNA replication over entry or egress. We therefore carefully re-checked our manuscript to ensure that we never "conclude" that Loqs is involved in "viral RNA replication" and only in "viral replication". We only "propose" the hypothesis that Loqs regulate vRNA regulation. Indeed, already in the R1 version of our manuscript, we were careful in our conclusions. E.g.:

- *In the abstract, lines 31-32* "We propose that DENV hijacks a factor of the RNAi mechanism for replication of its own RNA.*"*
- *In the results, lines 274-276* "we propose that DENV non-structural proteins recruit Loqs to viral replication organelles, where it interacts likely through its dsRNA-binding motifs with viral RNA to facilitate viral RNA replication."
- *The summary has now been corrected according to the reviewer's suggestion, now stating on line 44* "Loquacious functions as a proviral factor that is recruited to replication organelles to facilitate viral [~~RNA~~] replication".

Reviewer #3: N/A

Part III – Minor Issues: Editorial and Data Presentation Modifications

Reviewer #1: None noted

Reviewer #2:

- The new figure cited in the rebuttal and the main test „Fig S1d" is missing from the supplementary file.

Thank you. The main text was corrected to "Fig S2D", see line 217.

- The GFP empty control in Fig5 was required to control for bleedthrough of the 488 channel in the 568 channel rather than the colocalization. In general, resolution of Fig 5 (including the high-res downloadable file) is still very poor.

In absence of bleedthrough of the 488 channel into the 568 channel in the images of the mock infected cells, we do not deem it necessary to include an additional GFP empty control.

Although we agree that the resolution could be improved, the resolution of the current images is sufficient to support our conclusion that dsRNA and Loqs signal strongly colocalize. We therefore have not repeated the microscopy experiments.

Reviewer #3: N/A
