# Supplementary material for: Arbovirus-vector protein interactomics identifies Loquacious as a co-factor for dengue virus replication in Aedes mosquitoes
Source: PLoS Pathog. 2022 Sep 8;18(9):e1010329. doi: 10.1371/journal.ppat.1010329 (PMC9488832; doi:10.1371/journal.ppat.1010329)
Supplement: Attachment — Submitted filename: 20220608-Loqs_PLOSpath-Response_to_reviewers_rev5_submitted.docx [file ppat.1010329.s012.docx]

Nijmegen, June 09, 2022

Manuscript number D-22-00212

Dear editors and reviewers,

We thank the editors and reviewers for their supportive comments and important suggestions for our manuscript “Arbovirus-vector protein interactomics identifies Loquacious as a co-factor for dengue virus replication in *Aedes* mosquitoes”.

We have addressed the reviewers’ comments in the revised manuscript. Importantly, we now provide new data addressing the main concerns of the reviewers: effect of Loqs knockdown on infectious titers, reciprocal mass spectrometry to confirm interaction of Loqs with viral proteins, quantification of negative sense viral RNA, and Loqs phenotype in cells lacking Argonaute 2.

Please find enclosed the revised manuscript, one version with our changes highlighted in yellow as well as a clean version. Our responses to the reviewers’ specific comments can be found below (in blue, italic font).

With best wishes,

Ronald van Rij

**Part I – Summary**

Please use this section to discuss strengths/weaknesses of study, novelty/significance, general execution and scholarship.

**Reviewer #1:** This is a very nice manuscript where the authors perform a DENV NS1-NS5 protein interactome study in mosquito cells. The manuscript is well written and the figures are nicely assembled and easy to follow. Their findings demonstrate a role of Loqs during flavivirus infection of mosquito cells by 1) showing, most likely indirect, protein interations between Loqs and NS1 2) co-localization during infection 3) silencing of Loqs is detrimental to flavivirus infection 4) Loqs binds to the 3'UTR, and 5) the interactions function independently of RNAi. I have two specific comments below.

*We thank the reviewer for the supportive comments.*

**Reviewer #2:** In this manuscript, Besson and colleagues systematically profile the cellular interactomes of NS1 and NS5 - two important flaviviral proteins with functions in RNA replication, replication complex formation and viral RNA modification(s) – within C6/36 cells, an RNAi-incompetent Ae. albopictus cell line. Exploiting an ectopic expression system to drive expression of the entire sub-genomic DENV viral genome (NS1-NS5) under the control of the invertebrate polyUbiquitin promoter, they generate epitope-tagged variants of the viral polyprotein, carrying NS1- and NS5-FLAG-tagged variants for use in AP-LC-MS/MS experiments. Among the specific NS1 and NS5 interactors, 22 prioritized host factors were followed-up in silencing experiments to assess their functional relevance for viral replication, and one of them – the host protein Loquacious - was investigated further by immunofluorescence, isoform-mapping and dsRNA-binding experiments. Altogether, the authors propose that LoqRA interacts with the 3’UTR of DENV genome, via recruitment through the NS1 protein and plays an important pro-viral function independent from the RNAi pathway.

The study is well-written and timely, as it addresses understudied aspects of virus-host interactions in invertebrates. However, the wealth of publications on Loquacious role in flavivirus replication, as well as other studies on NS1 and NS5-host interaction networks in mosquitoes (cited by the authors in the manuscript) partially hamper the novelty of the study. Most importantly the main conclusions and novel findings (Loquacious plays a proviral role, independent of its role in RNAi, to support replication organelle formation) are only partially supported by experimental evidence as some experiments lack important controls (i.e. EMSA) or might be interpreted differently (i.e. Luciferase assays) as outlined below.

*We thank the reviewer for the insightful comments. We have taken the comments into account to improve our manuscript. See our point-by-point reply below for clarifications and additional experimental evidence in support of our conclusions.*

*We do not agree with the reviewer’s perceived lack of novelty.*

- *There are indeed several papers that studied Loqs or its orthologs in flavivirus infection. Haac et al. in 2015 (*[*https://doi.org/10.1093/nar/gkv152*](https://doi.org/10.1093/nar/gkv152)*) described the role of Loquacious (Loqs) in small RNA biology in mosquito cells, but they did not explore its role in viral infection. Olmo et al. in 2018 (*[*https://doi.org/10.1038/s41564-018-0268-6*](https://doi.org/10.1038/s41564-018-0268-6)*) described the role of Loqs2, an* Aedes *specific paralog of Loqs, in antiviral defense in mosquitoes, but that study did not explore the role of Loqs.
  Shivaprasad et al recently submitted a pre-print (*[*https://doi.org/10.1101/2021.12.03.471060*](https://doi.org/10.1101/2021.12.03.471060)*), which was published last month while our manuscript was under revision (*[*https://doi.org/10.1371/journal.ppat.1010163*](https://doi.org/10.1371/journal.ppat.1010163)*). This paper identified Loqs as a protein interacting with the dengue virus 3’UTR and, like our findings, the authors showed that it is a proviral factor for flavivirus replication in* Aedes *cells. Importantly, while the conclusions of Shivaprasad et al. correspond with our conclusions, the experimental approaches significantly differ. Moreover, our manuscript provides important additional data: we describe for the first time the interactome of NS1, NS5 and Loqs in mosquito cells, we performed a functional screening of 22 genes not tested before in this setting, we analyzed small RNA sequencing data in infected and* Loqs*-depleted cells, we analyzed viral replication in* Ago2 *and* Loqs *double knockout cells, and we provide* in vitro *evidence of a direct interaction between Loqs and DENV 3’UTR RNA.
  Together, our manuscript provides a significant body of additional evidence for the importance of Loqs in flavivirus infection of mosquito cells. The independent and complementary research by Shivaprasad et al and us mutually strengthens the conclusion that Loqs is a proviral factor in mosquitoes. Our manuscript is therefore an important contribution to the scientific literature, and we note that the PLOS policy on Complementary Research should provide a six-month scoop protection period. We discuss the Shivaprasad paper in relation to our findings in our revised manuscript on lines 289-295 and again refer to their work on lines 331-334 and line 362.*
- *We agree with the reviewer that several flavivirus interactome studies have recently been published: A ZIKV capsid interactome study in mosquitoes by Gestuveo et al. (2021) (*[*https://doi.org/10.1038/s41467-021-22966-8*](https://doi.org/10.1038/s41467-021-22966-8)*), a DENV NS1 interactome in humans by Hafirassou et al. (2017,* [*https://doi.org/10.1016/j.celrep.2017.11.094*](https://doi.org/10.1016/j.celrep.2017.11.094)*), a DENV NS5 interactome in humans by De Maio et al. (2016,* [*https://doi.org/10.1371/journal.ppat.1005841*](https://doi.org/10.1371/journal.ppat.1005841)*), a DENV NS1 interactome, in the absence of other non-structural proteins required to establish the macromolecular environment for viral replication by Caraballo et al (2021,* [*https://doi.org/10.1101/2021.09.01.458517*](https://doi.org/10.1101/2021.09.01.458517)*). The extensive study of Shah et al. (2018;* [*https://doi.org/10.1016/j.cell.2018.11.028*](https://doi.org/10.1016/j.cell.2018.11.028)*) described the interactome of all DENV proteins and a few ZIKV proteins in human cells as well as several viral proteins (capsid, NS2B3, NS4A, NS5) in mosquito cells. Notably, their DENV NS5 interactome identified only a single hit in mosquito cells. We thus note that all studies used different experimental approaches than the one we used and that DENV NS1 or NS5 interactome in the context of other viral proteins in mosquito cells is thus far lacking. We have cited these publications in our manuscript.*

**Reviewer #3:** The authors of this study report an interesting novel, and rather unexpected, pro-viral function for Loquacious (Loqs) in dengue virus (DENV) replication in cells derived from two important mosquito vector species, Aedes aegypti and Aedes albopictus. The findings are significant because they fundamentally change our view of the canonically antiviral functions of proteins functioning within the RNA interference (RNAi) pathway in insects. The work also advances our understanding of how molecular virus-vector interactions influence arthropod-borne virus (arbovirus) replication in mosquito cells, which is still a poorly developed area of research that has important implication for how significant arboviral pathogens are transmitted. The study is thorough and well executed, and uses a wide range of complementary approaches, including proteomics/interactomics and functional RNAi screens to characterise virus-vector interactions, which are then dissected in molecular detail using viruses, replicons and biochemical assays of RNA-protein binding. The manuscript is well written and the figures well presented and I believe the work makes a valuable and important contribution to the field.

*We thank the reviewer for the supportive comments.*

**Part II – Major Issues: Key Experiments Required for Acceptance**

Please use this section to detail the key new experiments or modifications of existing experiments that should be absolutely required to validate study conclusions.

Generally, there should be no more than 3 such required experiments or major modifications for a "Major Revision" recommendation. If more than 3 experiments are necessary to validate the study conclusions, then you are encouraged to recommend "Reject".

**Reviewer #1:**

1. The authors demonstrate a reduction in viral RNA, but does this translate to reduced infectious particles? It would have been nice to see plaque assay or focus forming unit data. Are samples still available that such assays could be easily performed?

*We thank the reviewer for this important comment (also raised by Reviewer #2, comment 3). We have performed the proposed experiment and observed that* Loqs *knockdown strongly reduced infectious viral titers in the culture supernatants (> 9- and 33-fold for DENV and ZIKV, respectively), confirming our original observations using viral RNA as a readout. The new experiment is shown in Fig. 4G and described on lines 210-216.*

2. While the authors did a lot of very nice work, I was left wondering if their cell culture observations were transferrable to mosquitoes. The impactfulness of this work would be significantly increased if the authors were able to observe decreased viral loads in mosquitoes upon Loqs suppression. However, the results might be a mixed bag because suppression of Loqs would also prosumably retard RNAi and, therefore, no differences might be observed. In lieu of performing mosquito experiments this might be an area the authors could speculate upon in the discussion section.

*We agree that it will be important to study the role of Loqs for DENV replication in mosquitoes* in vivo*. However, as noted by the reviewer, silencing of the RNAi pathway in whole mosquitoes brings a new degree of complexity and will require extensive additional experiments, and we thus deem it beyond the scope of the current manuscript. This was briefly discussed on lines 303-305.*

**Reviewer #2:**

1. It would be important to include control pull-downs using baits with a similar subcellular localization (i.e. an unrelated FLAG-tagged protein) rather than the untagged version of the polyprotein). Indeed, the specific distribution of the interactors in the volcano plots and the outcome of the statistical analysis directly reflects the use of “empty” pull-down as control. As a result, nearly all the viral proteins appear as specific interactors in both NS1 and NS5 dataset (Fig1), and both proteins share >30% of common host protein interactors (Fig2a) despite the rather divergent subcellular localization (NS1=ER vs. NS5=Cytoplasm+Nucleus).

*The proper choice of a control is indeed essential for quantitative, label-free mass spectrometry. We specifically chose to use untagged polyprotein rather than an unrelated FLAG-tagged protein, suggested by the reviewer. Since DENV infection and individual viral proteins modulate the proteome and transcriptome of the host (e.g. Chen et al, 2021,* [*https://doi.org/10.1038/s41598-021-03211-0*](https://doi.org/10.1038/s41598-021-03211-0)*; Chowdhury et al, 2021,* [*https://doi.org/10.3389/fitd.2021.708817*](https://doi.org/10.3389/fitd.2021.708817)*), expression of host genes is likely different in cells expressing the DENV polyprotein and cells expressing an unrelated FLAG-tagged protein. Due to the sensitivity of label-free mass spectrometry, these differentially expressed proteins will be identified as significantly enriched/depleted in the sample expressing DENV proteins, even if they do not form protein complexes with viral proteins. The use of an untagged control circumvents this problem and is therefore a better control than an unrelated protein.*

*We explain our choice for this control more clearly in the revised manuscript on lines 119-122 and lines 426-430.*

*The interaction of nearly all viral proteins with both NS1 and NS5 is expected as they form the replication complex that assembles in the replication organelles to replicate DENV RNA (Neufeldt et al., 2018,* [*https://doi.org/10.1038/nrmicro.2017.170*](https://doi.org/10.1038/nrmicro.2017.170)*). This also explains the overlap between NS1 and NS5 interactors, despite the differences in subcellular localization. Rather than reflecting nonspecific interactions, we interpret the overlap in the NS1 and NS5 interactomes as confirmation that we detected physiologically relevant multiprotein complexes.*

2. Along these lines, validation of the NS1-Loqs interaction upon infection (reciprocal IP using the available GFP-tagged Logs) would serve as important validation of the MS data.

*We agree that a reciprocal IP of DENV non-structural proteins with Loqs is important and we therefore performed the experiment suggested by the reviewer. We infected Aag2 Loqs-PA:GFP cells and analyzed Loqs-PA interactors by mass spectrometry. As expected, we observed a strong enrichment of RNAi factors Dicer1, Dicer2 and Ago1. Importantly, we found NS1 along with NS3 and NS5 to interact with Loqs, significantly enriched by at least 5.6-fold (p < 0.05). These new data are now included in the manuscript, lines 191-196, Fig. S1 and Table S4. The associated methods are on lines 384-385, 403-405, 438-444*

*We do not think that the interaction between Loqs and NS1 is a direct one, but most likely the Loqs-NS1 interaction is mediated by other viral proteins such as NS3 or NS5. Indeed, our data correlate with the recent paper from Shivaprasad et al (*[*https://doi.org/10.1371/journal.ppat.1010163*](https://doi.org/10.1371/journal.ppat.1010163)*), who detected an interaction of Loqs with NS3 by co-immunoprecipitation followed by western blot. We discuss this on lines 329-334.*

3. The readout of the silencing experiments and all downstream k.d. experiments exclusively relies on qRT-PCR on viral RNA, however it would be paramount to functionally validate at least the main host factors using an infectivity-based readout (i.e. TCID50, FFU or PFU assays). This is especially important as some experiments suggest a lack of Logs k.d. effect on virus replication (i.e. Fig4G, see comments below). Furthermore, standard deviations appear extremely high (i.e. Fig. 4E-H) and lack uninfected/NT-silenced controls.

*Thank you for this important point, which was also raised by Reviewer #1 (comment 1). We have performed the proposed experiment and confirmed in new titration assays that* Loqs *knockdown strongly affects viral titers of DENV and ZIKV. These data are now included in the manuscript in Fig. 4G and lines 213-215.*

*We agree that in some of the experiments error bars are somewhat large, but our results (*Loqs *knockdown vs viral RNA levels) are consistent between multiple experiments presented in the manuscript, as well as between multiple cell types, different viruses and different time points, altogether serving as independent confirmations. Moreover,* Loqs *knockdown experiments are now confirmed with* Loqs *knockout data, provided strong genetic support for the importance of Loqs for flavivirus replication. We are therefore confident of the validity of our conclusions. Uninfected controls were not deemed necessary as we analyzed the effect of Loqs on viral replication.*

4. The conclusion that Loqs proviral activity is independent of its RNAi regulatory function (result section; from row 209 onwards) is only indirectly supported by the data. It would be straightforward to compare the outcome of Logs silencing on virus replication in both RNAi-competent (U4.4) and RNAi-deficient cells (C6/36) already available and used by the authors in this manuscript.

*We agree with the reviewer that it would be nice to confirm this in cells deficient for both RNAi and Loqs (the same point was raised by Reviewer #3, Comment 2). In our hands, RNAi-mediated gene knockdown is inefficient in C6/36 cells. Therefore, we generated* Ago2*;* Loqs *double knockouts U4.4 cells using CRISPR/Cas9, containing out-of-frame mutations on all Loqs alleles.* Loqs *knockout in the background of* Ago2 *knockout strongly reduced viral replication of both DENV and ZIKV, even to undetectable levels for ZIKV. Importantly, the* Loqs *inactivation phenotype with this genetic knockout was stronger than our original observations using RNAi-mediated knockdown, providing strong support for the proviral phenotype of Loqs, independent of RNAi. These new data are now included in the manuscript, see Fig. 4L, Fig. S3, lines 244-251 and corresponding methods lines 389-395 and 594-614.*

5. The conclusion that Logs silencing does not affect RNA translation while inhibiting viral RNA replication is not experimentally supported. Indeed, experiments using the luciferase reporter virus do not show any reduction in luciferase activity upon Logs silencing, suggesting that both RNA replication and translation are not affected. If any, in U4.4 cells, silencing of Logs slightly enhances viral RNA replication (Fig 4G). Furthermore, this conclusion is not compatible with the authors’ hypothesis suggesting a role for Logs in viral RNA stability through binding of the 3’-UTR (one would expect to see a reduction here) and seemingly in contrast with qRT-PCR results presented in Fig. 3.

*We realize that the interpretation of the reviewer (and Reviewer #3, who raised a similar concern; Comment 1) assumes that the replicon assays measures translation of replicated viral RNA at later time points. Indeed, after transient transfection of a flavivirus replicon RNA, it is expected to observe a peak of luciferase translation from transfected viral RNA over the first 20-40h, whereas luciferase activity increases at later time points > 20-30 h) due to the translation of newly replicated viral RNA (e.g. Alvarez et al., 2015,* [*https://doi.org/10.1016/j.virol.2005.06.009*](https://doi.org/10.1016/j.virol.2005.06.009)*).*

*However, under our experimental conditions, we fail to observe the replication phase past the initial 20-40h in RNAi-competent mosquito cell lines. This was observed with several DENV replicons as well as a ZIKV replicon (see Fig. R1 below), and we found that a DENV replicon could only be stably maintained in RNAi-deficient mosquito cells (unpublished data). We currently do not understand the reason for this, but it seems that either transfection of mosquito cells with replicon RNA is inefficient, or that transfected RNA is efficiently cleared.*

*Therefore, our replicon assay only monitors translation of viral RNA, but cannot be used to assess viral RNA replication. In Fig. 4H (Fig. 4G in the original submission), luciferase expression from transfected DENV replicon RNA was not significantly different in dsLoqs and dsCTRL treated Aag2 cells, suggesting that Loqs does not affect RNA translation. In the revised manuscript, we provide a brief explanation of the limitation of the replicon assay on lines 220-223.*

*The difference in luciferase activity between dsLoqs and dsCTRL treatment in U4.4 cells was not statistically significant. As these replicon data from U4.4 cells did not provide any additional insights over the Aag2 cell data, we removed them from the revised manuscript.*

**Figure R1**. Expression of Nluc translated from a

ZIKV-Nluc replicon. Aag2 cells were transfected

with replicon RNA and protein extracts were

collected at the indicated times.

6. The RNA binding experiments (EMSA; Fig5A) would require an additional MAP-tagged protein as control to ensure specificity and accuracy of binding affinity measurements. Alternatively, the use of a non-flaviviral RNA might suffice. These aspects are particularly important as the input RNA of all the other control probes (5’UTR, NS5, NS1; see “free XX region”), as well as higher MW bands appearing at higher concentrations of purified protein (>200nM), appear significantly lower when compared to the corresponding 3’UTR.

*We thank the reviewer for this important comment and included the requested control experiments in the main manuscript. Specifically, we demonstrate that MBP:GFP does not have RNA binding activity, whereas MBP-Loqs-PA and PB efficiently bind dsRNA. The data are now presented in Fig. 6A-B and described on line 267 and lines 1078-1082.*

7. Result section – line 250: the hypothesis that Loqs is recruited to the replication organelles and binds viral RNA through its dsRNA-binding motif would require experimental confirmation. Indeed, in EMSA experiments only ssRNA has been used. (see comment above).

*The EMSA experiments were performed on folded ssRNA which is expected to form dsRNA structures, especially in the 3’UTR (Huber et al, 2019,* [*https://doi.org/10.1038/s41467-019-09391-8*](https://doi.org/10.1038/s41467-019-09391-8)*). Our experiments indicate that Loqs binds with high affinity to dsRNA as well as 3’UTR RNA, albeit that affinity for dsRNA is higher than for 3’ UTR RNA (Kd = ~2-20 nM vs ~50-60 nM, respectively, Fig. 6C, D).* *We deem it highly likely that the dsRNA binding domains mediate 3’ UTR binding, but we agree with the reviewer that we cannot formally exclude that other domains mediate binding to the 3’ UTR. We have therefore adjusted our statement, which now reads: “*Altogether, we propose that DENV non-structural proteins recruit Loqs to viral replication organelles, where it interacts, likely through its dsRNA-binding motifs, with viral RNA to facilitate viral RNA replication”*, lines 273-275.*

**Reviewer #3:**

1. I’m not fully convinced of the interpretation that Loqs affects viral genome replication but not viral RNA stability or translation. The data show that Loqs knockdown has no effect on transiently transfected DENV replicon, but does reduce viral RNA levels during infection. I agree that that the replicon data indicate that Loqs does not seem to affect viral RNA stability or translation, however, unless I’m mistaken this particular replicon also replicates its RNA, and therefore the replicon assay also indicates that Loqs does not affect viral RNA replication. The major difference between viral infection and the replicon assay is that the replicon bypasses viral entry and uncoating steps, and therefore it seems to me that Loqs rather affects these early stages of the viral life cycle rather than viral genome replication. I think the authors would need to perform additional experiments to support their current conclusions. For example, they could transfect the infectious viral RNA into cells and assess whether viral RNA levels are still reduced in the absence of Loqs when entry/uncoating is bypassed. Alternatively, one could measure the accumulation of negative-sense viral RNA in both the replicon and infection experiment to directly measure viral genome replication.

*In our original submission we failed to properly explain the limitation of our replicon assay. As also noted in response to Reviewer #2 (Comment 5), it is expected that in the early stages after transfection of replicon RNA (up to 20-30 h) luciferase is expressed by direct translation of the transfected RNA, while replication takes off later on. In mosquito cells, we have not been able to observe the luciferase peak that depends on viral replication, despite testing multiple DENV and ZIKV replicons. Apparently, transfection of replicon RNA is insufficiently efficient or replicon RNA is cleared before replication has been initiated. Therefore, our replicon assay only monitors translation of viral RNA, but cannot be used to assess viral RNA replication. Thus, our results from the replicon assay indicate that silencing of* Loqs *does not affect viral RNA translation. In the revised manuscript, we provide a brief explanation on lines 220-223*

*Considering that Loqs contains multiple dsRNA binding domains, we do not consider it likely for Loqs to be involved in the viral entry (or egress) process and we deem it much more likely that it is involved in the regulation of viral RNA. We agree with the reviewer that quantification of negative-strand would provide additional support for our conclusions. We therefore performed the experiment proposed by the reviewer and found that* Loqs *knockdown resulted in a strong decrease of viral negative sense RNA, providing further support that Loqs is important for efficient viral RNA replication. These new data are presented in the revised manuscript in Fig 4I, Fig. S3D, and lines 228-231. The associated methods are on lines 559-561.*

2. The authors also conclude that the pro-viral role of Loqs is independent of Loqs’s function in the RNAi pathway. The data shown does indeed indicate that Loqs knockdown does not affect the accumulation of virus-derived siRNAs. However, I do not think this is sufficient to support the claim that Loqs acts independently of the RNAi pathway. Evidence that might better support this claim would be to test the effect of Loqs knockdown in the presence of concurrent Dicer2 or Ago2 knockdown, or in Dicer2 or Ago2 CRISPR knockout cell lines (in which the RNAi pathway would not be functional). Another question I have is whether the authors saw differences in piRNA production in this experiment?

*Reviewer #2 raised a similar point (Comment 4). We agree that the original support for our conclusion was not sufficiently strong and we performed the experiment proposed by the reviewer. We inactivated the* Loqs *gene by CRISPR/Cas9-mediated editing in U4.4-Ago2^KO^ cells to obtain cells in which both* Loqs *and* Ago2 *are functionally inactivated. Viral RNA levels in these U4.4-Ago2 ^KO^; Loqs ^KO^ cells were much lower than viral RNA levels in U4.4-Ago2 ^KO^ cells. This experiment provides strong genetic support that the proviral effect of Loqs is independent of the RNAi pathway. This new experiment is now included in the manuscript, see Fig. 4L, Fig. S3 and lines 244-251, and the associated methods on lines 385-391, 590-610, 543-546.*

*We agree with the reviewer that it would indeed be interesting to study whether piRNAs were affected by* Loqs *knockdown. Our sequencing data suggest that viral piRNAs were not affected, but sequencing depth was not sufficient to draw robust conclusions and we therefore did not include this in the manuscript.*

3. In Fig 5, the dsRNA staining shows a lot of background in mock cells – which I suspect is due to the presence of other insect viruses persistently infecting these cells. Since the authors are investigating whether Loqs localises to DENV replication compartments, could they perform a sequence-specific stain for DENV RNA to specifically stain DENV replication compartments and reduce the background noise in the mock?

*The reviewer rightly notes the moderate dsRNA background signal in mock infected Aag2 cells (the same point was raised by Reviewer 2, minor comment 5). We deem it unlikely that this is due to persistent infections for two reasons. First, our Aag2 cells have been cleared of persistent RNA virus infections, including CFAV and PCLV (which we specify on lines 395-397). Second, background staining is observed particularly in the nucleus, whereas we expect CFAV and PCLV to replicate in the cytoplasm.*

*We expect that the dsRNA staining in the nucleus reflects sites of high production of (structured) RNA. Importantly, there is a clear difference in cytoplasmic dsRNA staining between mock and DENV-infected cells, and the nuclear background staining thus does not affect our conclusions. In the revised manuscript, we acknowledge the background staining in the nucleus on lines 258-260.*

**Part III – Minor Issues: Editorial and Data Presentation Modifications**

Please use this section for editorial suggestions as well as relatively minor modifications of existing data that would enhance clarity.

**Reviewer #1:** (No Response)

**Reviewer #2:**

1. Why use the C6/36 cells (RNAi-incompetent) as model system for the AP-LC-MS/MS study? The follow-up functional screen was then done in the RNAi-competent U4.4 cell line – why not directly use the U4.4 cell line?

*Indeed, the use of RNAi-competent cells for the MS experiment would have been ideal but based on preliminary experiments, the pPUb-NS constructs were expressed at significantly higher levels in the C6/36 cell line than in the RNAi competent U4.4 or Aag2 cells. For now, we have not explicitly explained this in the manuscript, but if the editor or reviewer deem it useful, we are happy to include an explanatory statement.*

2. In the method section, row 368: “cells were transfected with 10 mg”: probably meant 10 µg?

*This has now been corrected on line 415 to 30 µg of plasmid per flask.*

3. Supplementary figure S2 (Protein purification): the SDS-PAGE does not show a single band for the purified protein post dialysis, but rather a smear of 20-30 kDa for both Loqs-PA and PB. Could this reflect incomplete cleavage of the MAP-tag? This might affect interpretation of the EMSA experiments.

*MBP-Loqs was purified without cleavage of the MBP tag. This has now been made explicit in the labelling of Fig. 6. We acknowledge the smear – which could be due to some protein degradation or to a technical problem with this particular gel – but we do not think it affects the interpretation of the EMSAs.*

4. Supplementary Figure S4: oversaturated signals in IF images for both GFP-tagged proteins and dsRNA.

*Indeed, the uncropped images of Fig. S6 (previously Fig. S4) shows heterogeneity in expression of Loqs-GFP, with some cells in which the signal was saturated. Only cells with low to moderate expression were considered to interpret the subcellular Loqs localization, which has now been made explicit in the legend to Fig. S6 (lines 1137-1138).*

5. FIG 5: there is quite significant background for dsRNA in the mock sample. Furthermore, no “empty”-eGFP control was used in this panel.

*Please, see our response to Reviewer #3, who raised a similar point (Comment 3). In brief, the reviewer correctly notes that there is moderate dsRNA signal in the nucleus of mock infected Aag2 cells. However, this is not expected to affect our conclusions, as this staining is clearly different from the strong, cytoplasmic dsRNA staining of DENV infected cells.*

*We did not deem it necessary to include an empty-GFP control. Based on our experience, GFP shows a diffuse staining throughout the cytoplasm as well as in the nucleus in Aag2 cells (see Fig. R2 below). This staining is clearly distinct from the cytoplasmic foci formed by Loqs-GFP.*

**Figure R2**. Expression of GFP in Aag2 cells. Cells were transfected with a pAc-EGFP
plasmid and GFP expression was imaged. The nuclei were stained with Hoechst.

**Reviewer #3:**

1. In Fig 5, I found it quite difficult to see the visual differences that the authors describe in the localisation of Loqs, particularly any differences between the two isoforms. Could the authors provide some quantification here to strengthen these findings?

*We agree that the description of the perceived differences between the Loqs isoforms was insufficiently clear. Unfortunately, our visual observations proved difficult to quantify and we removed the description of the differences between the two isoforms from the revised manuscript. See line 260-261. Importantly, this does not affect the interpretation and conclusions in the manuscript.*

2. In the Materials and Methods, the authors should specify what (if any) corrections for multiple comparisons were applied in their statistical analyses.

*We thank the reviewer for this important comment. We clarified the use of one-way ANOVA for multiple comparisons in Fig. 3 and 4; lines 646-647, 1029-1030, 1063-1064.*

**Minor other changes:**

*1. We have removed the schematic outlines of the experiments from Fig. 4 and Fig. 5, as we (retrospectively) felt that they were not very informative and cluttered the figures.*

*2. Due to the inclusion of two new Supplemental figures and table (Fig. S1 and S3, Table S4), numbering of the supplemental figures has changed.*
